# Previous knowledge can induce an illusion of causality through actively biasing behavior

**DOI:** 10.3389/fpsyg.2015.00389

**Published:** 2015-04-08

**Authors:** Ion Yarritu, Helena Matute

**Affiliations:** Departamento de Fundamentos y Métodos de la Psicología, Universidad de DeustoBilbao, Spain

**Keywords:** previous knowledge, expectations, causal judgments, cognitive bias, causal learning, contingency learning, contingency judgment, illusion of causality

## Abstract

It is generally assumed that the way people assess the relationship between a cause and an outcome is closely related to the actual evidence existing about the co-occurrence of these events. However, people's estimations are often biased, and this usually translates into illusions of causality. Some have suggested that such illusions could be the result of previous knowledge-based expectations. In the present research we explored the role that previous knowledge has in the development of illusions of causality. We propose that previous knowledge influences the assessment of causality by influencing the decisions about responding or not (i.e., presence or absence of the potential cause), which biases the information people are exposed to, and this in turn produces illusions congruent with such biased information. In a non-contingent situation in which participants decided whether the potential cause was present or absent (Experiment 1), the influence of expectations on participants' judgments was mediated by the probability of occurrence of the potential cause (determined by participants' responses). However, in an identical situation, except that the participants were not allowed to decide the occurrence of the potential cause (Experiment 2), only the probability of the cause was significant, not the expectations or the interaction. Together, these results support our hypothesis that knowledge-based expectations affect the development of causal illusions by the mediation of behavior, which biases the information received.

## Introduction

Existing evidence questions the ability of humans to make accurate assessments of causality (e.g., Allan and Jenkins, 1983; Perales and Shanks, 2007; Hannah and Beneteau, 2009; Blanco et al., 2011; Matute et al., 2011). Even though there are conditions in which people are perfectly capable of making precise estimations about causal relationships between events (e.g., Ward and Jenkins, 1965; Peterson, 1980; Shanks and Dickinson, 1987; Allan, 1993), there are also well-established situations that invariably lead to a biased interpretation of the available evidence, resulting in systematic deviations from the objective assessment of causality (e.g., Jenkins and Ward, 1965; Allan and Jenkins, 1983; Blanco et al., 2009; Hannah and Beneteau, 2009). Importantly, those deviations may produce the illusion that two events are causally related when they are, in fact, totally independent from each other (e.g., illusions of causality such as the illusion of control; Langer, 1975; Alloy and Abramson, 1979; Matute, 1994, 1995; Yarritu et al., 2014). Some studies suggest that prior expectations might be at the basis of such deviations from the objective assessment of causality, and consequently, they are the main source of these illusions (e.g., Chapman and Chapman, 1967). The main purpose of the present study is to explore the role that expectations have in the development of the illusion of causality.

It is generally assumed that causal judgments are built on the basis of available evidence derived from the co-occurrence of cause and outcome events (e.g., Jenkins and Ward, 1965; Shaklee and Mims, 1981; Crocker, 1982; Allan and Jenkins, 1983; Kao and Wasserman, 1993; White, 2009; Shanks, 2010). Two events can be combined in four possible ways depending on their presence or absence. When the cause event is present, the outcome can be present or absent. Likewise, when the cause is absent, the outcome can also be present or absent. These four possibilities are commonly represented by the lower case letters, *a*, *b*, *c*, and *d*, as shown in Table 1 (e.g., Jenkins and Ward, 1965; Allan and Jenkins, 1983; Kao and Wasserman, 1993; Perales and Shanks, 2007). Recognizing the frequencies of each of these different cases, we can easily compute the probabilities of the occurrence of the outcome in the presence of the cause, *p* (*O*|*C*) = *a*/(*a* + *b*), and in its absence, *p* (*O*|¬*C*) = *c*/(*c* + *d*). The difference between these two conditioned probabilities leads to the contingency index, Δ*p* (Jenkins and Ward, 1965; Allan, 1980; Allan and Jenkins, 1983). Δ*p* is a mathematical representation of contingency that has been broadly taken as the normative value against which participants' judgments in human causal learning experiments should be compared (e.g., Jenkins and Ward, 1965; Shaklee and Mims, 1981; Allan and Jenkins, 1983; Shanks and Dickinson, 1987). Furthermore, the concept of contingency has been regarded as the cornerstone of most of the models proposed to explain causal learning (e.g., Rescorla, 1968; Rescorla and Wagner, 1972; Cheng, 1997).

**Table 1:** **Contingency table**.

		**Outcome**	
		**Present** (*O*)	**Absent** (¬*O*)
Potential cause	Present (*C*)	*a*	*b*
	Absent (¬*C*)	*c*	*d*

*C, potential cause; O, outcome*.

Evidence shows that human causal judgments are sensitive to contingency (e.g., Jenkins and Ward, 1965; Allan and Jenkins, 1983; Shanks and Dickinson, 1987). However, a number of studies present data showing that under certain situations deviations from normatively expected assessment are systematic (e.g., Alloy and Abramson, 1979; Allan and Jenkins, 1983; Kao and Wasserman, 1993; Hannah and Beneteau, 2009; Blanco et al., 2011; Matute et al., 2011; Matute and Blanco, 2014). In order to explain those deviations some researchers have suggested that people do not weigh the different cells of the contingency matrix shown in Table 1 in the same manner (e.g., Wasserman et al., 1990; Kao and Wasserman, 1993; Anderson and Sheu, 1995; White, 2009). Following this *cell-weighting hypothesis*, each of the four cells has a different relative weight with respect to the others, in such a way that some cells have a greater impact than others in the causal estimations. The typically observed order of cells based on their relative weight is *a* > *b* > *c* > *d* (see Wasserman et al., 1990; Kao and Wasserman, 1993). Following this view, the instances that have greater impact on causal judgments are those in which the occurrence of cause and outcome coincide (cell “*a*”). Supporting this idea, a number of studies have indicated the special role that coincidences have in the elaboration of causal judgments (Jenkins and Ward, 1965; Crocker, 1982; Matute et al., 2011). These studies have attributed the responsibility for deviations from the objective assessment to the special meaning conferred by people to coincidences. In that way, the conditions that contribute to a larger number of cases in which cause and outcome coincide should promote the overestimation of the relationship between them (see Yarritu et al., 2014).

This latter assumption concurs with existing evidence of biases that systematically lead to deviations in causal judgments, such as the *outcome*- and *cause-density* biases. These biases are related to the marginal probabilities of the outcome and cause events. A significant number of studies have shown that participants' judgments tend to be higher as the probability of the outcome, *p* (*O*), increases (e.g., Alloy and Abramson, 1979; Allan and Jenkins, 1983; Matute, 1995; López et al., 1998; Msetfi et al., 2005; Hannah and Beneteau, 2009; Byrom et al., 2015), even when that probability is the same in the presence and in the absence of the potential cause (i.e., zero contingency; e.g., Alloy and Abramson, 1979; Allan and Jenkins, 1983; Matute, 1995; Blanco et al., 2013). Similarly, it has been observed that as the probability of the cause, *p* (*C*), increases, participants' judgments also tend to increase (Allan and Jenkins, 1983; Perales and Shanks, 2007; Hannah and Beneteau, 2009; White, 2009; Musca et al., 2010; Vadillo et al., 2011), even when the potential cause and the outcome are non-contingently related (e.g., Hannah and Beneteau, 2009; Blanco et al., 2013; Yarritu et al., 2015). The combination of these two biases increases the overestimation of the causal relationship when the two probabilities, *p* (*C*) and *p* (*O*), are high (Blanco et al., 2013). Note that a high probability of both events necessarily leads to a large number of coincidences, which, as predicted by the cell-weighting hypothesis, should produce the overestimation of causality.

Therefore, how people weight the available evidence seems to determine participants' causal judgments, and produce, under certain conditions (e.g., situations in which the number of coincidences is high), deviations from the objective causal relationship. However, some researchers have proposed another source from which deviations of the objective causal assessments could appear: the person's previous knowledge-based expectations (e.g., Chapman and Chapman, 1967; Crocker and Taylor, 1978; Abramson and Alloy, 1980; Nisbett and Ross, 1980; Peterson, 1980). For instance, Chapman and Chapman (1967) found that when their participants were asked to judge the relationship between randomly paired diagnostic test signs and symptoms, they tended to illusorily correlate some of the signs with certain symptoms in a way that was congruent with their previous knowledge.

One of the very first researchers who pointed out the relevance of people's previous knowledge about causal relationships was Kelley (1972). Kelley used the concept *causal schemata* to refer to the body of knowledge that a person holds about how causes and outcomes covary in the environment. According to Kelley (1972), people act as naive scientists using in a correct manner the evidence from the covariation between events (i.e., cells of the contingency matrix), but eventually, when this source of information is weak or insufficient, they invoke causal schemata to solve the uncertainty. More recent approaches to this issue from the perspective of Bayesian networks suggest that people possess a *causal grammar* which, like Kelley's causal schemata, holds abstract principles about how events can be causally related, and provide hypothesis spaces in which the possible relationships can be tested (e.g., Tenenbaum et al., 2006, 2007).

Nevertheless, other authors have gone beyond Kelley's position suggesting that people's previous knowledge can be even more decisive than available evidence in the establishment of causal estimations (e.g., Nisbett and Ross, 1980; Metalsky and Abramson, 1981; Alloy and Tabachnik, 1984; White, 1989; Griffiths and Tenenbaum, 2005). In this vein, White (1989) proposed that the available evidence about the covariation between cause and outcome is taken into account only when the person considers the causal relationship to be plausible. Congruent with White's position, other researchers have suggested that causal inference has two main stages, one in which the causal structure is inferred from the available evidence (which would determine the plausibility of the relationship) and other one in which the strength of the relationship is estimated (e.g., Waldmann and Hagmayer, 2001; Griffiths and Tenenbaum, 2005; Lagnado et al., 2007; De Houwer, 2009). For example, Griffiths and Tenenbaum (2005) propose a causal inference model that distinguishes between the acquisition of a causal structure, in which the decision about the existence of the relationship is made, and the estimation of strength, in which the extent with which the events are related is estimated. These researchers assume that structure acquisition is more fundamental because the strength of the relationship can only be estimated once the structure is acquired. Following up on the idea that people must consider the existence of a relationship before they estimate its intensity, Fugelsang and Thompson (2000) carried out a series of experiments in which they manipulated the scenarios in which causes and outcomes were presented. Given the general knowledge of their participants, some relationships were constructed so they seemed plausible, whereas others were meant to be implausible. In addition, they manipulated the covariation evidence presented in each scenario varying the contingency between the cause and the outcome. Both previous knowledge and contingency between cause and outcome significantly affected participants' causal judgments. However, the effect of contingency was weaker when the scenarios presented implausible relationships. That is, in implausible situations, available evidence was not as influential as it was in the situations in which previous knowledge supported the relationship.

Therefore, these results suggest that previous knowledge may affect how available evidence is evaluated. Nisbett and Ross (1980) also pointed out a position congruent with this idea. They indicated that the information from previous knowledge could mitigate the influence of available evidence when this evidence was incongruent with such knowledge. They proposed that this occurs because previous knowledge influences how people collect, recall, and interpret new evidence.

Assuming this approach, Metalsky and Abramson (1981; see also Alloy and Tabachnik, 1984) suggested that when previous knowledge strongly suggests a particular covariation and available evidence is unambiguous but suggests the opposite conclusion, people must solve the incongruence. According to Alloy and Tabachnik (1984) this *cognitive dilemma* tends to be solved in favor of previous knowledge. Interestingly, these authors propose how this inclination toward previous beliefs could occur. They suggested that people distort the evidence they encounter by overweighting those instances that are consistent with their previous beliefs and underweighting those which are inconsistent. In that way, the estimations about the frequency of each different instance, i.e., each one of the four contingency cell types described above (*a*, *b*, *c*, and *d*), will depend on the person's previous expectations about the relationship between the cause and the outcome. For example, if a person believes that there is a relationship between a cause and an outcome, he/she will tend to overestimate the frequency of the cases that confirm the relationship (i.e., cells *a* and *d*) and underestimate the frequency of instances that disconfirm the existence of a relationship (i.e., cells *c* and *b*). If, on the contrary, a person believes that the relationship does not exist, he/she will tend to overestimate the frequency of disconfirming cases and underestimate the frequency of confirming cases. Similarly, Crocker and Taylor (1978) propose that in order to assess the relationship between events, people will tend to establish *a priori* hypotheses based on their previous knowledge. The sense of these hypotheses will determine the degree of confidence given to each instance of information received, conferring greater credit to cases that confirm those hypotheses than to those that refute them. Similar assumptions can be found in causal models based on Bayesian networks (e.g., Waldmann and Martignon, 1998; Griffiths and Tenenbaum, 2005) through the introduction of *prior probability distributions* in causal inferences. This prior probability distribution formalizes the person's *a priori* beliefs about the plausibility of different hypotheses before the observation of new evidence, but taking into account their background knowledge. In this way, a person's prior beliefs can favor some hypotheses over alternative ones and can affect how this person will interpret new evidence.

Therefore, people seem to have a tendency to interpret available evidence in favor of their hypotheses, which are based on their previous knowledge. This position is similar to the assumptions of the *confirmation bias* research tradition. Confirmation bias is a phenomenon by which people tend to seek or interpret available evidence in a way that is congruent with their beliefs, expectations, or *a priori* hypotheses (see Nickerson, 1998 for a broad review). This confirmation bias usually implies not only a biased way of interpreting available evidence, but also an active behavior consisting on seeking the information that confirms previous hypothesis (e.g., Wason and Johnson-Laird, 1972; Klayman and Ha, 1987; Evans, 1989; Nickerson, 1998). In this line, some authors have suggested that previous knowledge may affect the way people make interventions (as an opposite to merely observe) to find the evidence that they need to make causal inferences (e.g., Steyvers et al., 2003; Waldmann and Hagmayer, 2005; Schulz et al., 2007). This all suggested to us that when people have the opportunity to intervene over the evidence they receive, prior expectations might affect their behavior, and, in consequence, might bias the information to which they are exposed. If this is true, previous expectations could affect the development of the illusion of causality by actively biasing behavior (and therefore the information that people receive). The present research tests this idea.

## Overview of the experiments

In a typical causal learning experiment participants are exposed to information about the co-occurrence of causes and outcomes, and then they are asked to emit their judgments about the relationship between the two events (e.g., Jenkins and Ward, 1965; Allan and Jenkins, 1983; Wasserman, 1990). This information is usually presented in a trial-by-trial format in which each trial represents one of the four contingency cell types described above (Jenkins and Ward, 1965; Wasserman, 1990). The participants of this type of experiment can play a passive or an active role in the task (see Yarritu et al., 2014). That is, participants can passively observe in each trial whether an external stimuli acting as a potential cause is present or absent (e.g., a medicine that has been administered or not to a fictitious patient) and then they can observe whether or not the outcome occurs (e.g., whether the patient recovers or not). Alternatively, they can be requested to make an active response that acts as a potential cause (e.g., to administer or not the medicine to the patient) and then observe whether the outcome occurs or not given their action (e.g., whether the patient recovers or not). In the first case, in which the participants have a passive role, they have no way of influencing the information they receive. The sequence of trials (i.e., the frequency and order of each instance of the four contingency cells) is pre-programed by the experimenter. In the second case, however, the participants determine through their responses the presence or absence of the potential cause in each trial (e.g., they choose whether or not to administer the medicine to the patient). This means that the proportion of instances in which the potential cause occurs, *p* (*C*), is subject to the free choice of the participants who perform the experiment. Even though the experimenter programs the outcome to occur with a given probability, and this is independent of the participants' behavior, the participants do influence, to a certain degree, the information they are exposed to. If they respond with high frequency they will receive more *a* and *b* instances than *c* and *d* instances, whereas if they respond with low frequency, they will receive fewer *a* and *b* instances than *c* and *d*.

We have already mentioned that *p* (*C*) is a powerful determinant in the development of illusions of causality (e.g., Perales and Shanks, 2007; Hannah and Beneteau, 2009; Blanco et al., 2013). Importantly, this effect appears regardless of whether the potential cause is an external event or is the participants' behavior (see Hannah and Beneteau, 2009; Yarritu et al., 2014 for a discussion on this topic). For instance, in her study about the illusion of control, Matute (1996) found that participants tended to respond with high frequency [i.e., high *p* (*C*)], in order to obtain a desired outcome, and thus, they developed a high illusion of control. When instructed about the need to respond with less frequency her participants did reduce their *p* (*C*) and were able to accurately detect that they had no control over the outcome. This effect has been found in many situations in which, in one way or another, participants have been induced to respond differently. For example, in Yarritu et al.'s (2014) study, participants were restricted in the number of responses they could make; in Hannah and Beneteau's (2009) work, participants were told, in each trial, how and when to respond; in Byrom et al.'s (2015) study, participants were asked to try to respond with a particular frequency. In this way, the illusion of causality was reduced. Therefore, what could we expect when a person is free to respond to obtain a desired outcome? We propose that under these circumstances people's previous expectations about the causal relationship will influence how they respond. If participants believe that their responses (i.e., cause present) will produce the desired outcome, they will tend to respond with high probability (in order to obtain the outcome, and perhaps also in order to confirm their expectations; see General Discussion). If, on the contrary, participants believe that the outcome will occur when no response is given (i.e., cause absent), they will tend to respond with low probability.

Thus, we suggest that by inducing different expectations we should be able to induce different patterns of behavior, which in turn could lead to a biased exposure to information. This biased exposure to information will yield a deviation of the objective assessment of contingency, which ultimately should lead to the illusion of causality. That is, we propose a mediational hypothesis by which expectations should affect participants' assessments of causality by modifying their *p* (*C*). In order to test this hypothesis we conducted two experiments in which the previous expectations of the participants were manipulated, (a) in a free active response situation (Experiment 1); and (b) in a passive situation in which *p* (*C*) was manipulated by the experimenters (Experiment 2). The first experiment was designed to test the mediational hypothesis that the manipulation of expectations leads to differences in the judgments of causality through an effect on *p* (*C*). The second experiment was designed to control the potential effect of the expectations over both, the *p* (*C*) and the judgments of participants, which would compromise the mediational hypothesis. We anticipated that previous expectations would modulate the response patterns of participants, which in turn would determine *p* (*C*) and, in consequence, the degree of illusion of causality developed by the participants.

## Experiment 1

In this experiment we used a computerized trial-by-trial learning task in which the mission of participants was to learn about the effectiveness of a fictitious medicine to cure the crises caused by a fictitious disease. Participants were free to administer or not the medicine to a fictitious patient in each trial, and then they observed if the patient had recovered or not. Half of the participants were informed that the previous recovery rate for patients who had taken the medicine was 80% (8/10). The other half were informed that the recovery rate for the patients who had not taken the medicine was 80% (8/10). This recovery rate was the same under both conditions and coincided with the probability of patients' recovery throughout the experimental task, regardless of whether or not the participant had been administered the medicine. This means that the medicine was totally ineffective, as the probability of patients' recovery was independent of its intake. At the end of the experiment participants were requested to make a judgment about the effectiveness of the medicine. In sum, the computer program collected the participant's response in each trial as well as the judgment requested at the end of the experiment. These variables were what we needed to test a mediational model: expectations (independent variable), *p* (*C*), i.e., the participants' response probability during the experiment, defined as the number of trials which included at least one response divided by the total number of trials (mediator variable), and judgments about the effectiveness of the medicine emitted at the end of the experiment (dependent variable). Because there was no contingency between the cause and the outcome, the correct causal judgment should be zero. Of particular interest for the present research is whether different groups develop different degrees of illusion (overestimation) of causality, and whether this effect is mediated by differential patterns of behavior.

### Method

#### Participants and apparatus

Fifty-one anonymous Internet users who visited our virtual laboratory (http://www.labpsico.deusto.es) participated in the experiment. In agreement with ethical guidelines for human research through the Internet (Frankel and Siang, 1999) we did not ask them for any data that could compromise participants' privacy, nor did we use cookies or software in order to obtain such data. The procedure was approved by the ethics committee of the University of Deusto. The experimental task was developed in a HTML document dynamically modified with the use of JavaScript. This technology allowed running the experiment through an Internet browser in such a way that volunteers could participate anonymously on their personal computers. The software randomly assigned each participant to one of the two experimental groups, resulting in 26 participants in the group with expectations of cause-outcome relationship (ExC-O Group) and 25 participants in the group with expectations of no cause-outcome relationship (ExNoC-O Group).

#### Procedure and design

The procedure used in the present experiment was an adaptation of the allergy task (Wasserman, 1990). The reason we used this task is that it has been widely used in studies of human causal learning, including those focusing on the illusion of causality (e.g., Yarritu et al., 2014). In addition, the task has been successfully tested in Internet experiments (e.g., Matute et al., 2011). Using the initial instruction screens, all participants were prompted to imagine being a medical doctor who specialized in a rare disease called “Lindsay Syndrome.” They were then told that there was a medicine called “Batatrim” that could cure the crises produced by the disease, but that this medicine was still in the testing stages. They also received information about the actual frequency with which the outcome occurred, but this information was worded differently for each of two groups. One group of participants was informed about the frequency with which the patients recovered from the crises (outcome) when they had taken the medicine (potential cause). The other group was informed about the frequency with which patients recovered when they had not taken the medicine. The exact wording of this sentence (translated from Spanish) was (a) *The first trials with this medicine showed that of 10 patients who had taken Batatrim, eight recovered from the crises for Group ExC-O*, and (b) *The first trials with this medicine showed that of 10 patients who had NOT taken Batatrim, eight recovered from the crises for Group ExNoC-O*.

Thus, this sentence was the only difference between the two groups and was our experimental manipulation. Note that the two informative sentences are complementary descriptions of the actual situation that participants will be exposed to: a situation in which there is no relationship between the medicine and recovery but the probability of spontaneous recovery is high. In both groups the outcome (recovery) occurs in 80% of the trials regardless of whether the cause is present or absent. The purpose of our subtle instructional manipulation was that participants in the ExC-O Group would start the experiment with the expectation that the medicine would produce the recovery of the patients, whereas participants in the ExNoC-O Group would start the experiment with the expectation that the medicine was not necessary to produce that cure. As the entire experimental task was identical for both groups of participants, any difference between the behaviors shown by the two groups must be attributed to the expectations invoked by this slightly different sentence. The remaining instructions informed participants that they would see a series of records of patients, each representing a patient to whom they could administer Batatrim to observe if the patient recovered from the crisis or not.

Once the participant had read the instructions, the experiment began. It consisted of two parts, a training phase and a test phase. In the training phase, participants observed, trial by trial, medical records of fictitious patients. There were 100 trials (patients) in total. In each trial participants were asked whether or not they wanted to administer the medicine to the patient. The response was made by clicking one of two buttons, *Yes* or *No*. Once the choice was made, participants could see if the patient had recovered or not from the crises. Therefore, the presence or absence of the potential cause (the medicine intake) was the participant's choice. The outcome (patient's recovery from the crises) was presented in some trials following a pre-programmed pseudorandom sequence. That is, the patients' recovery occurred independently of the participant's decision to dispense the medicine. The contingency between the medicine intake and the patients' recovery was null, and the administration of the medicine neither increased nor decreased the probability of recovery. The programmed probability with which the outcome occurred was 0.8. As mentioned earlier, such high probabilities of the outcome tend to favor the development of the illusion of causality (e.g., Alloy and Abramson, 1979; Allan and Jenkins, 1983; Matute, 1995). After completing all 100 training trials, participants began the test phase in which the following question was presented (translated from Spanish): *To what extent do you think that Batatrim was effective in healing the crises of the patients you have seen?* The answers were given by clicking on a 0–100 scale, anchored at 0 (*definitely NOT*) and 100 (*definitely YES*).

### Results and discussion

#### The effect of expectancies on participants' behavior

**Figure 1:**
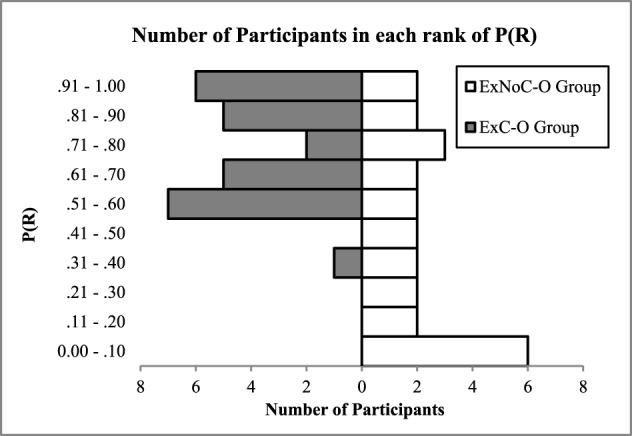
**Distribution of response probabilities of participants in each group for Experiment 1**.

Figure 1 shows the distributions of the *p* (*C*), i.e., the probability of responding in both groups. As seen in the figure, the two groups responded differently. Participants who were prompted to believe that the administration of the medicine produced the improvement of the patients (ExC-O Group) tended to dispense the medicine in most trials; virtually all of the participants had response rates beyond 50%. Moreover, a considerable number of them responded in more than 90% of trials (including four participants who responded in every trial). On the other hand, the distribution of response probabilities of the participants who were prompted to believe that the patients' recovery occurred often among those who did not take the drug (ExNoC-O Group) was more heterogeneous. The modal tendency was to respond below the 10% of trials (three participants responded in none of the trials). However, whereas the ExC-O Group are certainly responding at high rates the ExNoC-O Group have much larger variability, with a much less consistent trend, which suggest an asymmetric effect of expectations over the response rates of the two groups. Means (and standard errors of the means) of the probability of response of both groups can be seen in Table 2. A simple *t*-test^1^ comparing both groups confirmed that the probability of responding in Group ExC-O was significantly higher than in Group ExNoC-O, *t*_(49)_ = 3.83, *p* < 0.001, *d* = 1.09.

**Table 2:** **Mean judgments and probabilities of response [i.e., *p* (*C*)] in Experiment 1**.

**Group**	**Judgment**		***p* (*C*)**	
	***M***	***SEM***	***M***	***SEM***
ExC-O	65.153	4.923	0.737	0.037
ExNoC-O	45.160	6.611	0.448	0.066

*M, mean; SEM, standard error of the mean*.

**Figure 2:**
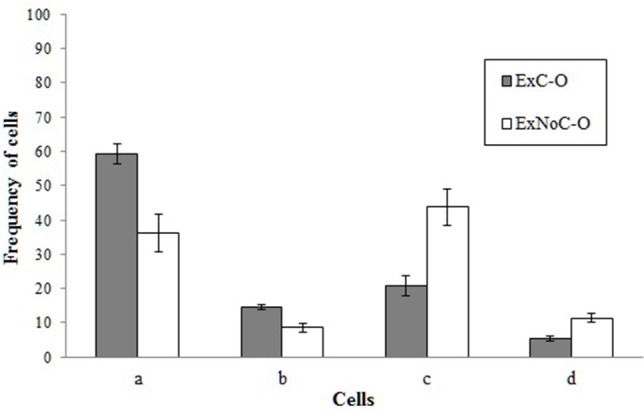
**Mean frequencies of the four contingency cells to which the participants of each group were exposed to during the Experiment 1**. Error bars represent the standard error of the mean.

These response tendencies led participants to miss important information about one of the two possible states of the potential cause. Whereas participants who administered the medicine in almost every trial (or in all of them) had little evidence (or no evidence at all) about what happens when the patients do not take the medicine (cause absent), those who administered the medicine in almost none of the trials had little evidence about what happens when patients take the medicine (cause present). In order to further understand the differences that their differential behavior caused in the information to which participants were exposed in each group, we decided to analyse the frequencies of the four contingency cells that they experienced. The means of these frequencies for each group can be seen in Figure 2. Four *t*-tests comparing the two groups confirmed that the frequencies were different between both groups for cell *a*, *t*_(49)_ = 3.76, *p* < 0.001, *d* = 1.07; for cell *b*, *t*_(49)_ = 3.87, *p* < 0.001, *d* = 1.11; for cell *c*, *t*_(49)_ = 3.76, *p* < 0.001, *d* = 1.07; and for cell *d*, *t*_(49)_ = 3.87, *p* < 0.001, *d* = 1.11. These results testify the magnitude of the difference in the information to which each group was exposed, revealing their biased exposure to information.

Table 2 also shows the mean (and standard error of the mean) of the participants' judgments of effectiveness for the two groups. We expected that our manipulation of the expectations would affect not only the participant's response rates but also the judgments given at the end of the experiment. A *t*-test comparing the two groups confirmed our hypothesis, with judgments being significantly higher in the ExC-O Group than in the ExNoC-O Group, *t*_(49)_ = 2.44, *p* = 0.018, *d* = 0.7.

Since participants were free to respond in each trial and the occurrence of the outcome was predefined in a pseudorandom sequence, there was some degree of variance in the contingency to which participants were actually exposed. However, it cannot be assumed that this variance was different between the groups, *t*_(49)_ = 1.872, *p* = 0.07, *d* = 0.53. For this reason, we doubt that differences between groups in other variables are due to the variance of the actually experienced contingency^2^.

#### Mediational effect

**Figure 3:**
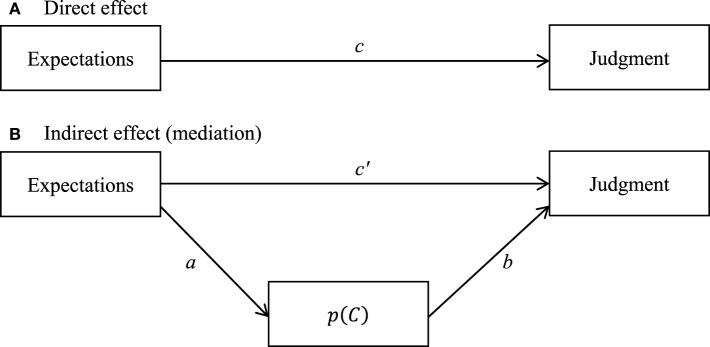
**Mediational model tested in Experiment 1. (A)** Direct effect of the expectations over the judgments. **(B)** Indirect effect in which *p* (*C*) acts as a mediator in the relationship between the expectations and judgments.

We expected that the differences in the probability of responding [i.e., probability with which the potential cause occurs, *p* (*C*)] would explain the differences between judgments. In other words, we expected that *p* (*C*) acted as a mediator variable in the effect of the manipulation of expectations over participants' judgments. To test this hypothesis we carried out a mediational analysis. In order for a given variable [*p* (*C*)] to be considered as a mediator between an independent variable (expectations) and a dependent variable (judgments), three criteria must be present (see, e.g., Baron and Kenny, 1986): (a) the independent variable must significantly predict the dependent variable (path *c* in Figure 3A); (b) the independent variable must significantly predict the mediator variable (path *a* in Figure 3B); and (c) the mediator variable must significantly predict the dependent variable, once controlled for the effect of the independent variable (path *b* in Figure 3B). In addition, if the effect of the independent variable over the dependent variable decreases to zero after the inclusion of the mediator variable in the model (i.e., absence of significance of path *c* in Figure 3B), it is said that there is a perfect mediation. Table 3 shows the results of the mediational analysis conducted using the PROCESS procedure developed by Hayes (2013) which implements bootstrap confidence intervals for inference about indirect effects. As shown in the table, all three criteria necessary to be considered a mediational variable were met. In addition, Table 3 shows that the influence of expectations on judgments vanished as *p* (*C*) was included in the model. This means that this variable is acting as a mediator between the expectations and the judgments, and that this is a perfect mediation. A Sobel test (Sobel, 1982) conducted over this model showed that the mediation effect was significant (*z* = −3.14, *p* < 0.001). The effect size of the mediation effect (as computed by the product of the effect sizes of the paths *a* and *b*) was large (*dr* = 0.67).

**Table 3:** **Results of the mediational analysis of Experiment 1**.

**Predictors**	***B***	***ETB***	**β**	***t***	**Targets**
Step 1: expectations	−19.994	8.198	−0.329	−2.439^*^	Judgment
Step 2: expectations	−0.2897	0.0757	−0.480	−3.827^**^	*p* (*C*)
Step 3: expectations	−0.470	7.390	−0.008	−0.064	Judgment
*p* (*C*)	67.395	12.238	0.670	5.507^**^	Judgment

*B, non-standardized coefficient; ETB, standard error of B; *p* (*C*), potential cause probability*.

* *p < 0.05*.

** *p < 0.001*.

These results support the mediational hypothesis that previous expectations about a relationship between a potential cause and an outcome affect the assessment of causality between them by modifying the behavior of participants, which ultimately determines the probability with which the cause occurs in a free response task. However, given these data, it remains possible that the previous expectations affected both the judgments of the participants and the probability with which they decided to administer the medicine during the training trials. The design of Experiment 1 is not able to discard this hypothesis given that the two potential factors, expectations and *p* (*C*), were not controlled independently. In addition, given that *p* (*C*) was not manipulated, the causal role of this factor over the differences in judgments cannot be assumed. An experimental design that intervenes orthogonally over *p* (*C*) in addition to over the expectations is necessary to elucidate the real effect of each of these two factors over the judgments of participants.

## Experiment 2

Experiment 2 was designed to address the potential limitations of Experiment 1. Keeping in mind this objective, we now manipulated orthogonally the two factors involved in the mediational model proposed in Experiment 1, the previous expectations and the *p* (*C*). The learning task used in this second experiment was the same as the one used in Experiment 1. However, the new task demands a passive role of participants, instead of an active role. A passive learning task was used to allow for the experimental control of the instances in the contingency cell that the participants would receive in each trial, or in other words, in order to manipulate *p* (*C*). In order for Experiment 2 to resemble Experiment 1 as closely as possible the two levels of *p* (*C*) were programmed to be high (0.8) and medium (0.5), as these two values were similar to the mean probabilities displayed by the two groups in Experiment 1 (see Table 2). In line with the mediational hypothesis, we expected that once we controlled *p* (*C*) by depriving participants of the ability to respond freely, the effect of the previous expectations would disappear, showing uniquely the main effect of the probability of the potential cause.

### Method

#### Participants and apparatus

The participants were 114 anonymous Internet users who visited our virtual laboratory (http://www.labpsico.deusto.es). Data was treated in the same manner as in the previous experiment in respect to privacy and anonymity of participants. The experimental task was a version of the one previously used in Experiment 1, and it used the same technology. The software randomly assigned each participant to one of four experimental groups. This procedure resulted in 55 participants who were placed in Group ExC-O, 29 of whom were in the high *p* (*C*) condition and 26 of whom were in the medium *p* (*C*) condition. The other 59 participants were placed in Group ExNoC-O, 32 of whom were in the high *p* (*C*) condition and 27 of whom were in the medium *p* (*C*) condition.

#### Procedure and design

The computer program used for Experiment 2 was a version of that used in Experiment 1. As in the previous experiment, the manipulation of the expectations was conducted through instructions. Half of the participants were induced to expect that the medicine produced the improvement of the patients, whereas the other half was induced to expect that the medicine was not necessary to produce such improvement. Differently from the previous experiment, the task used in Experiment 2 did not allow participants to decide whether or not to administer the medicine. In each trial they could see whether the patient had taken Batatrim or not. At this point, they were requested to respond to a Yes/No predictive question (*Do you think the patient will recover from the crisis?*) The predictive question was introduced in order to maintain the participants' attention and to make sure they were reading the screen. Once the response was given, the participants could see whether the patient had recovered from the crisis. The training phase contained 100 trials. The sequence of potential cause (medicine intake) and outcome (recovery from crisis) pairings was pre-established in a manner that resembled the co-occurrence information received by the two groups in Experiment 1. Half of the participants were presented with a sequence of trials in which *p* (*C*) was of 0.8, which resembles the mean *p* (*C*) of Group ExC-O in Experiment 1. The other half was presented with a sequence of trials in which this probability was 0.5, which resembled the mean *p* (*C*) of Group ExNoC-O in Experiment 1. The probability of the outcome was high (0.8) in all cases. Likewise, the contingency was set to zero for all participants and we looked for differences in their illusion (overestimation) of causality. Therefore, Experiment 2 resulted in a between-subjects 2 × 2 design in which both expectations and *p* (*C*) were manipulated orthogonally. At the end of the training phase, the test phase presented participants with the same judgmental question as in Experiment 1, which was answered in an identical way.

### Results and discussion

**Figure 4:**
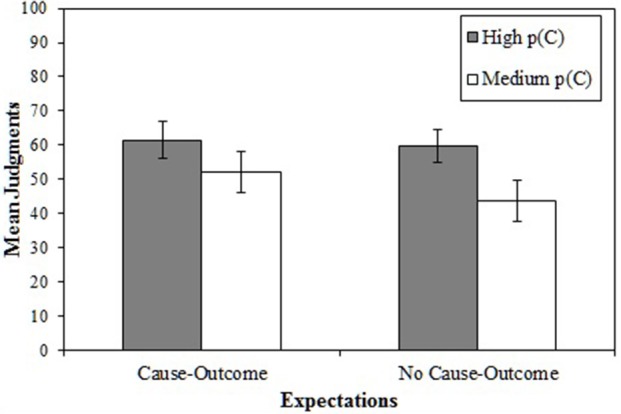
**Mean judgments in Experiment 2 for each condition of expectations and for each condition of *p* (*C*)**. The error bars represent the standard error of the mean.

Figure 4 shows the mean judgments in Experiment 2. As expected, the judgments of participants who were exposed to a high *p* (*C*) were higher that the judgments of participants who were exposed to a medium *p* (*C*). Moreover, mean judgments did not differ as a function of the expectations. A 2 × 2 ANOVA on participants' judgments as dependent variable showed a main effect of the probability of the potential cause, *p* (*C*), *F*_(1, 110)_ = 5.25, *p* = 0.024, η^2^_*p*_ = 0.05. Neither the main effect of the expectations nor the interaction were significant [largest *F*_(1, 110)_ = 0.83].

These results provide evidence that supports the mediational hypothesis, as they suggest that when the effect of the previous expectations over participants' behavior [*p* (*C*)] is controlled, their effect on the participants' judgments disappears. Only the effect of *p* (*C*)] remained significant. The results of this second experiment suggest that previous expectations do not affect the judgments. By contrast, only the information to which participants were exposed was actually affecting their judgments.

## General discussion

The results support the hypothesis that people's previous expectations about the causal relationship between two events affect their assessment of that relationship by modulating their behavioral patterns when they have the opportunity to decide about the presence, or absence, of the potential cause (Experiment 1). On the contrary, when the presence of the potential cause is not under the participants' control (Experiment 2), the influence of their previous expectations becomes overshadowed by that of the evidence to which they are exposed. This leaves causal judgments determined only by this last source of information, and therefore affected by well-known biases in contingency learning, such as the cause-density bias.

The results of the mediational analysis conducted in Experiment 1 suggest that the probability with which the cause occurred [i.e., *p* (*C*)]; in this case, the probability with which the participant acted] was affected by our manipulation of expectancies, and this behavior was able to explain the causal judgments emitted at the end of training. Moreover, the analysis showed that when the *p* (*C*) was controlled, the effect of the expectations over the judgments disappeared. The results of Experiment 2 strengthen the mediational hypothesis. They show that when the experimental setting does not allow participants to decide whether or not the potential cause is present, the effect of previous expectations over causal judgments (as well as the interaction), become not significant. As could be expected, however, the effect of the *p* (*C*) manipulation was significant. This means that when previous expectations could not influence the behavior of the participants, their effect on judgments became insignificant. Only through the mediation of behavior did previous expectations influence the development of the illusion of causality.

In Experiment 1, we anticipated that because participants in Group ExC-O believed that the medicine produced recovery, they would respond with higher probability than participants in Group ExNoC-O. The results confirmed this prediction. Interestingly, this could occur through at least two different motivations. On the one hand the participants might be motivated to obtain the outcome (patients' recovery); on the other one, they might be using a positive test strategy to confirm their hypothesis. In any case, this aspect was identical for both groups and therefore it cannot explain the differences observed in their behavior and judgments. The critical aspect of the present results is that they show that expectations affect the participants' response rates [i.e., *p* (*C*)], which may lead to exposure to biased information, and that this in turn will lead to misperceive the causal relationship. Experiment 2 provided further support for this idea. By providing a situation in which *p* (*C*) was preprogramed by the researchers, expectations could no longer influence judgments by producing changes in *p* (*C*) In this case, the potential influence of expectations on judgments was overshadowed by *p* (*C*), which now affected judgments directly.

The results of these experiments are, in part, congruent with the idea that people's previous knowledge and expectations can determine the treatment that they give to available evidence in the assessment of causal relationship (Crocker and Taylor, 1978; Nisbett and Ross, 1980; Metalsky and Abramson, 1981; Alloy and Tabachnik, 1984; Waldmann and Martignon, 1998; Griffiths and Tenenbaum, 2005, 2009). Along this line, some authors have suggested that previous knowledge may define how people interpret the available evidence, for example, by influencing the confidence given to each type of evidence (Crocker and Taylor, 1978) or by affecting the perception of the frequency of each of them (Alloy and Tabachnik, 1984). Our study suggests that the influence of previous expectations takes place before the evidence is interpreted, in a stage in which the evidence is not yet available. In a sense, expectations determine which information will be available. This seems congruent with the idea of prior probability distributions as they intervene in causal inferences before the presentation of new evidence (e.g., Waldmann and Martignon, 1998; Griffiths and Tenenbaum, 2005). Moreover, some researchers have suggested that these prior probability distributions, based on previous knowledge can also determine people's interventions on new evidence (e.g., Steyvers et al., 2003; Schulz et al., 2007). Consistent with this idea, in our experimental setting previous knowledge influenced the participants' behavior.

On the other hand, the approach claiming that previous knowledge is more decisive than available evidence in the establishment of causal estimations (e.g., Nisbett and Ross, 1980; Metalsky and Abramson, 1981; Alloy and Tabachnik, 1984; White, 1989; Griffiths and Tenenbaum, 2005; Lagnado et al., 2007) is not supported by the results of the present research. Following this approach, some authors suggested that evidence on the co-occurrence of two events is not taken into account when previous knowledge points toward the inexistence of a relationship between them (e.g., White, 1989; Griffiths and Tenenbaum, 2005). Similarly, Fugelsang and Thompson (2000) found an interaction between the effect of previous expectations and the available evidence. They suggested that the effect of this last factor was smaller when previous knowledge signaled that the causal relationship was unbelievable than when it was believable. Our results do not show an interaction between previous expectations and available evidence. Furthermore, there was not a main effect of previous expectations in our research when the available evidence about co-occurrence between the two events was controlled for.

The difference between our results and those of Fugelsang and Thompson (2000) may reside in the manner in which the two studies manipulate previous expectations. In our experiments the cover story was the same for all participants and recreated a plausible situation in which a potential relationship existed between a cause and an outcome (a medicine that cured the crises of a certain disease). In this situation we manipulated only the partial (and veridical) information that the participants received about the relationship before they started the experiment. To do aim, we changed just one sentence in the instructions of each group. One group was informed about the actual probability of the outcome in the presence of the cue, the other one was informed about the actual probability of the outcome in the absence of the cue. The Fugelsang and Thompson's study, however, manipulated the cover story so that some stories were about commonly believable relationships and others about unbelievable relationships. The fact that a relationship was seen as totally unbelievable could have led participants of Fugelsong and Thompson's study to leave the available evidence unattended. Our expectations manipulation, by contrast, was intentionally subtle, as we were aiming to test how minor differences in the way in which contingency information is presented might produce different expectations that might promote different judgments of causality. Interestingly, this manipulation of expectancies was able to affect the participants' judgments through behavioral changes, but not directly. The effect of expectancies on contingency learning was overshadowed by the available evidence when behavioral changes where neutralized.

The results of the present study are congruent with the approach which claims that the assessment of causality is mainly based on how people interpret the perceived evidence derived from the co-occurrence of cause and outcome events (e.g., Crocker, 1982; Wasserman et al., 1990; Kao and Wasserman, 1993; Anderson and Sheu, 1995; Perales and Shanks, 2007; White, 2009; Matute et al., 2011; Yarritu et al., 2014). According to this perspective, people assess each instance in the contingency cell in a different manner, being the instances in which the potential cause and the outcome coincide (i.e., cells *a*) the ones which typically most influence the estimation of causality (e.g., Kao and Wasserman, 1993). It follows from this assumption that any circumstance that contributes to a larger number of *a* instances, with respect to the other ones, will promote an overestimation of causality, which can lead to an illusion of causality in the conditions in which the potential cause and the outcome are actually unrelated (Yarritu et al., 2014). One of these circumstances is related to the probability with which the potential cause occurs. As mentioned in the Introduction, it has been shown that when the *p* (*C*) is high, causal judgments tend to be higher than when this probability is low (e.g., Perales and Shanks, 2007; Hannah and Beneteau, 2009; Matute et al., 2011; Blanco et al., 2013). The results of Experiment 2 show that, minor differences in this probability can determine the degree of illusion of causality.

In sum, this study shows how previous knowledge and expectancies can contribute to the development of causal illusions. Previous knowledge, or previously received information that was accepted as valid, biases behavior by increasing the probability of the potential cause (in this case, the participants' behavior). This biased behavior produces a cause-density bias, which is known to have an important role in the development of such as illusions.

One important implication from this research is that our results could explain, for example, how people who hold a favorable view about a pseudoscientific and ineffective medical treatment could bias themselves by taking the innocuous drug frequently and ignoring what might happen had not them taken it. This behavior could lead them to erroneously confirm their previous false belief. Importantly, however, our research also suggests that this bias could be attenuated by a simple advertising campaign informing people about what happens when the potential cause is not present. Our research shows that this information results in these people changing their behavior and therefore reducing their illusion as well.

### Conflict of interest statement

The authors declare that the research was conducted in the absence of any commercial or financial relationships that could be construed as a potential conflict of interest.

## Footnotes

^1^At first glance the distributions of *p* (*C*) depicted in Figure 1 seems not to fit to a normal distribution, which suggests that non-parametric statistic should be preferred to compare these two groups. For this reason, we conducted the Kolmogorov-Smirnov probe to test the normality of the two samples. The results indicate that we cannot refute the hypothesis of normality of the two group's *p* (*C*) distributions (*p*-values equal to 0.70 and 0.82 for the ExC-O group and the ExNoC-O groups, respectively). Given these results we decided to report the *t*-test comparisons. Nevertheless, we also conducted an alternative analysis using the non-parametric Mann-Whitney test and the results do not differ from those reported in the main text, *U*(49) = 163.00, *Z* = 3.06, *p* = 0.002.

^2^Participants with extreme response rates, those who responded in every trial or in none of them, lacked one of the two conditional probabilities needed to compute the experienced contingency (see Introduction), because they were not exposed to trials of one of the potential cause conditions (i.e., presence or absence of the medicine). Data from these participants could have been discarded as missing data. However, we decided to consider the conditional probability as zero in these cases, and compute the experienced contingency in that way, because those participants were highly representative of the response bias we were studying. Nevertheless, we also conducted an alternative analysis discarding the data of those participants. The results of this alternative analysis do not differ from the analysis presented here.
